# Superior Sagittal Sinus Thrombosis Complicating Typhoid Fever in a Teenager

**DOI:** 10.1155/2012/201203

**Published:** 2012-11-27

**Authors:** P. O. Okunola, G. E. Ofovwe, M. T. Abiodun, C. P. Azunna

**Affiliations:** Department of Child Health, University of Benin Teaching Hospital, Benin City 300001, Nigeria

## Abstract

Cerebral venous sinus (sinovenous) thrombosis (CSVT) is a rare life-threatening disorder in childhood that is often misdiagnosed. CSVT encompasses cavernous sinus thrombosis, lateral sinus thrombosis, and superior sagittal sinus thrombosis (SSST). We present an adolescent girl who was well until two weeks earlier when she had a throbbing frontal headache and fever with chills; she later had dyspnoea, jaundice, melena stool, multiple seizures, nuchal rigidity, and monoparesis of the right lower limb a day before admission. Urine test for *Salmonella* typhi Vi antigen was positive, and Widal reaction was significant. Serial cranial computerized tomography scans revealed an expanding hypodense lesion in the parafalcine region consistent with SSST or a parasagittal abscess. Inadvertent left parietal limited craniectomy confirmed SSST. She recovered completely with subsequent conservative management. Beyond neuropsychiatric complications of Typhoid fever, CSVT should be highly considered when focal neurologic deficits are present.

## 1. Introduction

Cerebral venous sinus (sinovenous) thrombosis (CVST) is a rare life-threatening disorder in childhood that is often misdiagnosed [1]. Its incidence varies between 0.4 and 0.7 per 100,000 per year, with more than 40% occurring in the neonatal period [1]. CSVT encompasses three basic syndromes: carvenous sinus thrombosis, lateral sinus thrombosis, and superior sagittal sinus thrombosis [2, 3].The mechanisms involve in the manifestations of CVST include the occlusion of cerebral veins or dural sinuses leading to cerebral oedema, parenchymal damage, or haemorrhage [1, 4]. Also, this can result in impaired cerebrospinal fluid (CSF) absorption via the arachnoid villi in the superior sagittal sinus [4]. Common aetiologic factors of CSVT in children include perinatal insults, dehydration, sepsis, connective tissue disorders, and prothrombotic state like antithrombin III, protein C, or protein S deficiencies [1, 3]. Septic thrombosis most commonly occurs in the carvenous sinus because its trabeculated irregular structure readily entraps bacteria seeding from facial and sinus infections [2, 5]. Septic superior sagittal sinus thrombosis usually follows meningitis or sinusitis caused by *Streptococcus pneumoniae*, *Staphylococcus aureus* or *Klebsiella* species [2].

CSVT in children often presents acutely with encephalopathy, headaches, or focal neurologic deficits, but subacute features like anorexia or lethargy may occur depending on the extent of cerebral parenchymal lesion and sites(s) of occluded sinus and veins [3, 6]. In carvenous sinus thrombosis, ocular signs like proptosis, chemosis, and ophthalmoplegia predominate; in lateral sinus thrombosis, headaches and intracranial hypertension are frequent, with or without aphasia and focal neurological deficits [5–7]. Conversely, superior sagittal sinus thrombosis usually presents with motor deficit and seizures, while isolated intracranial hypertension is rare [2, 6].

The diagnosis of CSVT is confirmed based on a combination of an abnormal magnetic resonance imaging (MRI) signal in a venous sinus and a corresponding absence of flow on MR venography [4, 5]. Computed tomography (CT) shows typical evidence of CSVT in about one-third of cases [3, 4]. Other relevant investigations include screening for prothrombotic disorders [3, 6]. The mainstay of therapy of septic dural sinus thrombosis is culture-guided high-dose intravenous antibiotics, but anticoagulation, steroids and surgery are appropriate in selected cases [2–4]. CSVT-specific mortality is less than 10% in uncomplicated cases [1, 4]. However, septic superior sagittal sinus thrombosis with extensive parenchymal damage can be highly fatal [2]. Coma is a predictor of death in childhood CSVT, and neurologic sequelae may be present in up to 40% of survivors [1, 6]. 

Although several neurologic complications of Typhoid fever have been described, CSVT is rarely reported even in large series in Typhoid-endemic regions [8]. Therefore, we present this case to highlight the occurrence of this unusual complication of enteric fever.

## 2. Case Report

 A 13-year-old girl (O.J.) was referred from a private clinic to the children Emergency Room of the University of Benin Teaching Hospital on July 11, 2011 (Hospital number 541303). She was a fourth-year student in a public secondary school and the eldest of 3 children living with parents in an urban slum in Benin City.

 She was apparently well until two weeks earlier when she had a throbbing frontal headache and fever with chills. The headache was distressing, but there was no photophobia, preceding trauma, or history of migraine. Cough, constipation, neck stiffness, and right leg weakness occurred second week of the illness, while jaundice, melena stool, and multiple seizures occurred a day before admission. There was no history of amenorrhoea or use of oral contraceptive pills. Her haemoglobin genotype is AA.

 Physical examination revealed an ill-looking fair-complexioned adolescent girl with rose spots on the trunk. She was dyspnoeic, jaundiced, pale, and febrile (T-40.2°C) but not dehydrated. She was well-grown for age.Neurological examination revealed full consciousness, normal fundoscopic findings, monoparesis of the right lower limb and nuchal rigidity. Pulse rate was 120 beats per minute, blood pressure was 130/70 mmHg, and chest findings were consistent with bilateral lower lobe consolidation. Further systemic examinations were unremarkable.

**Figure 1:**
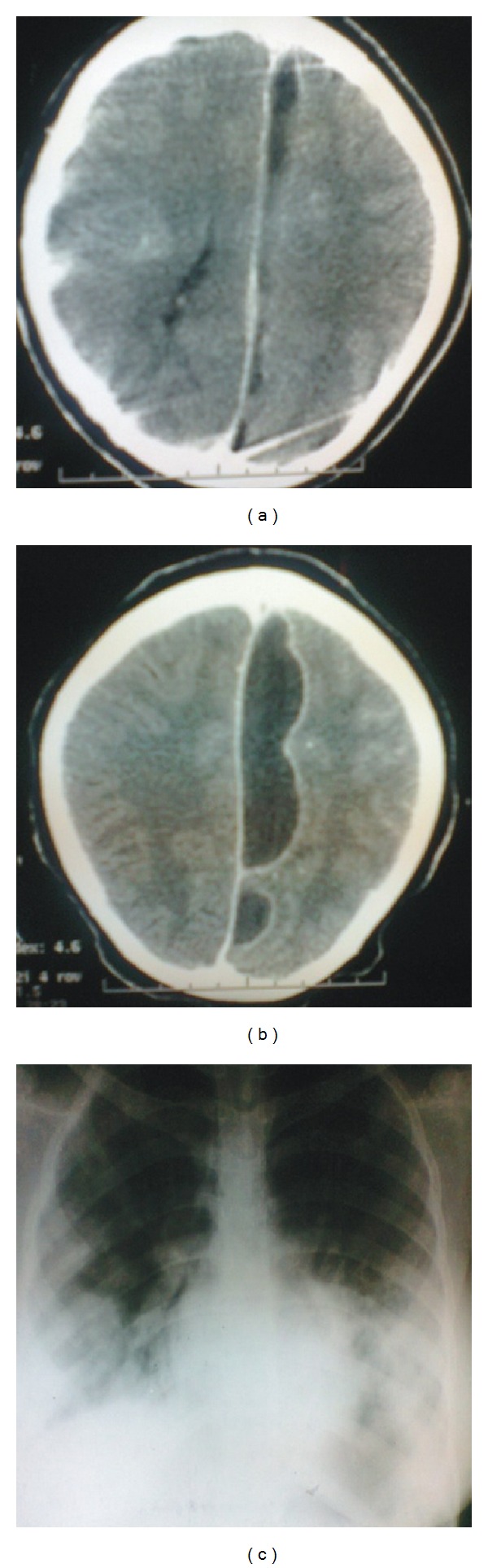
(a) Cranial CT scan of the patient showing a hypodense lesion in the parafalcine region with rim enhancement and effacement of the anterior of horn and body of the ipsilateral ventricle, consistent with superior sagittal sinus thrombosis or a left-parasagittal abscess; (b) a repeat scan 4 weeks later confirmed a marked progression of the lesion without significant shift of midline structures; and (c) CXR (AP view) shows bilateral mid and lower lobe opacities consistent with pneumonia.

 The initial assessment was Typhoid septicemia with a left-sided cerebral abscess. Cranial computerized tomography (CT) scan (Figure 1(a)) revealed hypodense lesion in the parafalcine region suggestive of left-parasagittal abscess, but superior sagittal sinus thrombosis could not be excluded. Chest X-ray confirmed bibasal lobar pneumonia (Figure 1(c)), and abdominal ultrasound showed bilateral acute renal parenchymal disease. Urinalysis showed numerous pus cells and cysteine crystals. Urine test for *S*. typhi Vi antigen was positive, and Widal reaction was significant. Blood, stool, and urine cultures yielded no growth. Serologic tests for viral hepatitis B and C were negative. Lupus erythematosus (LE) cell test was negative. Haematocrit was 25%, WBC-4.4 × 10^3^ cell/ul, and platelet-79 × 10^3^ cells/ul. CSF analysis was normal, and retroviral screen was negative. She received intravenous ceftriaxone  100 mg/kg/day, Metronidazole 7.5 mg/kg/dose 8 hourly, and supportive therapies.

 Based on the worsening of her neurological deficit into a right hemiparesis and the enlarging parasagittal lesion on repeat CT scan (Figure 1(b)) by the fourth week on admission, left parietal limited craniectomy was done revealing a tense bluish dura. Test aspiration yielded superior sagittal venous blood without evidence of intracerebral abscess. Haemostasis was secured. She improved on physiotherapy and other supportive postoperative management. She was ambulating without support at discharge 7th week on admission. She is on neurology and ophthalmology followup at the outpatient department.

## 3. Discussion

Cerebral sinovenous thrombosis in children is a rare under recognized disorder due to its wide range of clinical manifestations and diagnosis requiring specialized neuroimaging techniques [4, 5]. Our patient presented with headache, seizures, and a focal neurologic deficit, and alongside a CT scan lesion consistent with superior sagittal thrombosis fulfilling, the diagnostic criteria for CSVT in the International Classification of Diseases, 9th revision, clinical modification [9]. The inadvertent neurosurgical intervention enabled direct visualization of the thrombosed superior sagittal sinus. In this case, haematologic gastrointestinal, pulmonary, and urinary features are due to the multisystemic manifestations of the underlying Typhoid fever [10]. 

CSVT has been associated with the use of oral contraceptive pills in females, but our patient had no prior exposure to hormonal contraception [1]. Aetiologic agents of septic dural sinus thrombosis often reflect the primary source of infection [2, 7]. We confirmed *S*. typhi in this case based on the positive *S*. typhi Vi antigen in her urine and significant antibody titres against O and H antigens of *S*. typhi on Widal test. Negative cultures are apparently due to prior antibiotic therapies at the referring clinic. 

Septic CSVT may occur in Typhoid fever due to persistent secondary bacteraemia and cytokine secretion by macrophages during the symptomatic phase of this Gram-negative sepsis [10, 11]. Although the rarity of CSVT in Typhoid fever negates this assertion, this may be due to underdiagnosis, differences in inoculums sizes/*S*. typhi strains' virulence, or varied hosts' response due to differences in genetic constitutions [11]. Identified risk factors for septic CNS complications in Typhoid fever include endocarditis, sinusitis, pneumonia, and osteomyelitis of the skull [10, 11]. Our patient had bibasal lobar pneumonia increasing her risk of septic CSVT. Large case studies are required to fully determine the pattern of involvement of the Cerebral sinovenous system in septic thrombosis in Typhoid fever. Moreover, the exact pathogenesis of neuropsychiatric manifestations in typhoid fever is unclear, but suggested mechanisms include neuroendotoxin interactions, altered immune response, hyperpyrexia, dehydration, and electrolyte abnormalities [10, 11]. The index patient had no significant biochemical, fluid, or electrolyte derangement to account for her neurological features. 

Eradication of the offending organism is essential in septic sinus thrombosis in children and surgical drainage of abscess(es) may be indicated as attempted in this case [2, 7]. Anticoagulation and thrombolysis in children with septic superior sagittal sinus thrombosis can increase the risk of extensive intracerebral haemorrhage [2, 3]. The susceptibility of *S*. typhi to the third generation cephalosporin in children and prompt control of seizure, as well as physical and occupational therapies enhanced complete recovery in our patient. Subsequent recanalization of the thrombosed sinus and improved collateral circulation portend good outcome [4, 12]. Similarly, the absence of an underlying prothrombotic state, extensive cerebral damage, or coma in this case conferred good prognosis.

Beyond neuropsychiatric complications of Typhoid fever, CSVT should be highly considered when focal neurologic deficits are present.
